# Improving the Quality of Dissimilar Al/Steel Butt-Lap Joint via Ultrasonic-Assisted Friction Stir Welding

**DOI:** 10.3390/ma15051741

**Published:** 2022-02-25

**Authors:** Yu Chen, Fenghe Zhang

**Affiliations:** 1 School of Mechanical Engineering and Automation, Northeastern University, Shenyang 110819, China; crainy11@126.com; 2 Jiangsu Jianghua Valves Co., Ltd., Taizhou 225500, China

**Keywords:** friction stir butt-lap welding, ultrasonic vibration, dissimilar Al/steel joint, microstructural evolution, mechanical performance

## Abstract

A dissimilar AA7075/Q235 butt-lap joint was fabricated via ultrasonic-assisted friction stir welding (UaFSW), and the characteristics of the UaFSW joint were investigated systematically. The acoustoplastic effect of the ultrasonic vibration led to the softening of the materials and enhanced the material flow during welding, decreasing the volume of welding defects in the nugget zone of the UaFSW joint. With the help of ultrasonic vibration, a smooth and thin intermetallic compounds (IMCs) layer could generate along the Al/steel interface at the top of nugget zone, which possibly consisted of Al_5_Fe_2_ and Al_13_Fe_4_ phases. However, the positive effects of the ultrasonic vibration were weakened at low temperatures; consequently, the IMCs layer became discontinuous at the bottom of the nugget zone and the welding defects also formed. The ultrasonic vibration accelerated the dynamic recrystallization and refined the microstructures in the nugget zone due to the increased strain rate and stored energy. As a result, the UaFSW joint exhibited a better mechanical performance in comparison to the FSW joint, and the increment in the peak tensile load/elongation was more than twice. In addition, the UaFSW joint failed in the nugget zone along with the Al/steel interface, and the fracture mode was a mixture of ductile and brittle.

## 1. Introduction

Currently, Al/steel hybrid components have been widely applied in the transportation system, aimed at reducing both the weight and the fuel consumption of vehicles [1,2]. However, fabricating the dissimilar Al/steel joint is still full of challenges due to the differences between physical and chemical properties [3,4]. Especially, the limited solid solubility of aluminum and steel leads to the formation of brittle intermetallic compounds (IMCs), and the excessive growth of IMCs deteriorates the mechanical performance of Al/steel joints [5]. Friction stir welding (FSW) is a solid-state joining method characterized by the absence of melting [6,7], and the growth of IMCs can be inhibited due to the relatively low heat input of FSW. Up to now, numerous researchers have tried to join aluminum and steel together by FSW, proving the efficiency of FSW in producing the dissimilar Al/steel structure: Anaman et al. [8] produced a sound joint using AA5052 and DP 1200 steel, increasing the hardness of the FSW joint. Elnabi et al. [9] successfully joined AA1050 and low-carbon steel by FSW, observing the formation of IMC layers using different welding parameters. Liu et al. [10] joined dissimilar AA6061 and ASTM A36 steel without welding defects, analyzing the effects of FSW conditions on the Al-Fe interfacial transition layer development. Wang et al. [11] friction stir welded AA5083 and HSLA-65 steel and built the relationship between the thickness of IMC layers and the tensile properties of the FSW joint.

As mentioned above, the IMCs layer forms at the Al/steel interface during FSW, and moreover, the strength of the joints varies depending on the thickness of the IMC layer [11,12]. The formation of an IMC layer is necessary for strengthening the joints because the sudden transition in the chemical composition at the Al/steel interface can be weakened. Meanwhile, the thickness of IMC layers should be no more than 8 μm, as the cracks initiate and propagate easily through the brittle IMC layers [13]. In order to obtain thin IMC layers, the high-temperature period during FSW should be shortened [14]. However, at the same time, the material flow of Al/steel may become insufficient, resulting in the formation of welding defects [15]. Recently, a modified FSW technology called ultrasonic-assisted friction stir welding (UaFSW) is proposed by Ji et al. [16] and Wu et al. [17], which can be an effective way to elevate the welding quality of a dissimilar Al/steel joint further.

The positive effects of UaFSW on the welding quality can be outlined based on references [18,19,20,21,22]: Liu et al. [18] butt welded AA2024 with UaFSW and stated that the application of ultrasonic vibration reduced the time delay of the material flow, eliminating the tunnel defects. Hu et al. [19] detected a similar phenomenon during the UaFSW of an AA2219 butt-joint, and they illustrated the enhanced material flow attributed to the ultrasonic-induced formation of high-density vacancies. Padhy et al. [20] investigated the microstructural evolution of an AA6061 butt-joint made by UaFSW, finding that the ultrasonic vibration promoted the recrystallization process and led to better grain refinement in the nugget zone (NZ). Ji et al. [21] enhanced the plastic deformation and mixture of AA6061/AZ31 via UaFSW and, as a result, the tensile strength of the UaFSW joint was increased and the fracture mode of the joint shifted from brittle to ductile. Yu et al. [22] fabricated a dissimilar AA6061/Ti-6Al-4V lap-joint using UaFSW and reported that the interfacial diffusion of Al/Ti was accelerated, and the enlarged bonding area increased the shear load of the lap-joint.

So far, UaFSW is mainly utilized to elevate the welding quality of butt- and lap-joints, while the data on the butt-lap joint is scarce. Compared with the simple butt- or lap-joint, fabricating the butt-lap joint becomes more difficult because of its asymmetrical structure. Currently, the butt-lap joint is widely used in the train industry [23], and the demand for the dissimilar Al/steel butt-lap joint is also increasing. Thus, it becomes necessary to elevate the quality of the Al/steel butt-lap joint. In this study, the dissimilar Al/steel butt-lap joint was fabricated via UaFSW with the purpose of: (I) broadening the application range of UaFSW and (II) revealing the effects of UaFSW on the dissimilar Al/steel butt-lap joint.

## 2. Materials and Methods

**Figure 1:**
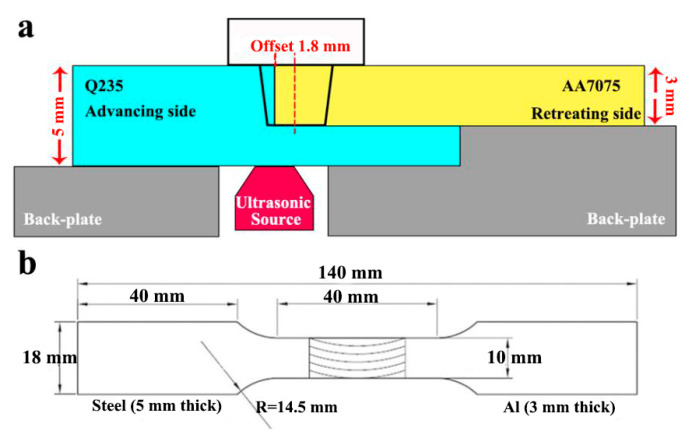
The schematics of (**a**) UaFSW and (**b**) tensile specimen used in the present work.

Rolled AA7075 and Q235 steel were chosen as the base metal (BM) in this work, and the nominal chemical compositions of AA7075/Q235 can be seen in Table 1. The thickness of AA7075 was 3 mm while that of Q235 was 5 mm. For AA7075, the initial temper after rolling was natural aging, and then the solution heat treatment was applied: firstly, soaking at 480 °C for 1 h and then air-cooling down to the room temperature. Figure 1a shows the schematic of UaFSW: a butt-lap surface was prefabricated on the Q235 plate and placed on the advancing side, the milling machine was applied to remove 3 mm steel from the top of Q235 plate and only left 2 mm on the bottom of Q235 plate. Meanwhile, the ultrasonic vibration with a 1000 W power output was exerted on the bottom surface of Q235. The W-Re stir tool with a flat shoulder of 18 mm diameter and a conical pin of 3.0 mm length was employed. The diameter of pin root and tip are 6 and 4 mm, respectively. The stir tool was shifted toward AA7075 and the offset of stir tool was 1.8 mm. The welding parameters kept constant for both FSW and UaFSW: 600 rpm rotational speed, 100 mm/min welding speed, 0.2 mm plunge depth, and 2° tilt angle.

In order to analyze the microstructural characteristics of dissimilar Al/steel butt-lap joints, optical microscopy (OM; Olympus-DSX-500, Tokyo, Japan), scanning electron microscopy (SEM; FEI-Quanta-600, Portland, OR, USA), and electron back-scattered diffraction (EBSD; FEI-Quanta-600, Portland, OR, USA) were employed. For OM observation, the FSW and UaFSW joints were mechanically polished and etched: Q235 was etched by the nitric acid–ethanol solution (the volume fraction of HNO_3_:C_2_H_5_OH was 1:24) and AA7075 was by the Keller’s reagent (the volume fraction of HF:HCL:HNO_3_:H_2_O was 2:3:5:90). Differently, the samples for SEM analysis were only mechanically polished in order to avoid the falling out of IMCs layer and second-phase particles caused by etching. The type of SEM detection was the secondary electron. Both the grain size and the recrystallization fraction of Al in the NZ were counted using EBSD technique, and the EBSD samples were electro-polished using the perchloric acid–ethanol solution (the volume fraction of HCLO_4_:C_2_H_5_OH was 1:9), and the scanning step of EBSD selected here was 0.36 µm. Besides, transmission electron microscopy (TEM; FEI-Tecnai-G20, Portland, OR, USA) was also used to observe the second-phase particles in the matrix of AA7075. The TEM samples were jet electro-polished at −25 °C with the nitric acid–methanol solution (the volume fraction of HNO_3_:CH_3_OH was 7:3).

The FM-700 Vickers hardness tester (HV; FUTURE-Tech, Tokyo, Japan) was employed to measure the distribution of hardness through the whole joint; the tested line was along the mid-thick of upper AA7075 plate, which started from Q235 at the advancing side and ended in AA7075 at the retreating side. Each tested line contained 30–40 measured points and the distance between the adjacent measured points was 0.5 mm. The test load and dwelling time are 50 gf and 5 s, respectively. Moreover, the mechanical performance of joint was evaluated by the tensile test (TS; Instron-5969, Beijing, China). The tensile specimens were produced perpendicular to the welding direction with a gauge of 40 mm length and 10 mm width (as shown in Figure 1b). The crosshead speed during tensile test was kept as 1.5 mm/min, and the peak tensile load of each joint was tested three times for average. The specification for the tensile specimen used is ISO 6892:1998.

## 3. Results and Discussion

**Figure 2:**
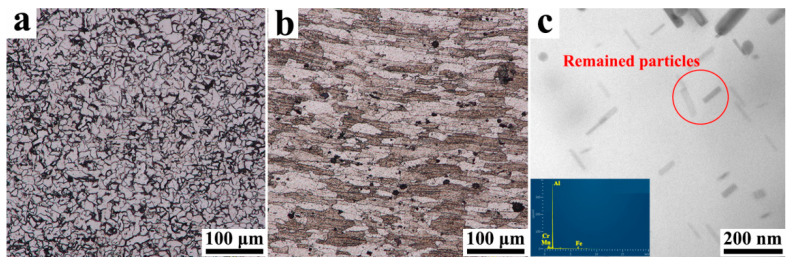
The grain morphologies in the BMs of (**a**) Q235 and (**b**) AA7075; (**c**) the remaining particles in the matrix of AA7075.

As the typical low-carbon steel, ferrite and pearlite are the main constituents of Q235 [24], thus the irregular ferrite grains can be observed in the steel matrix and the pearlite is located along the boundaries between the ferrite grains (Figure 2a). Compared with Q235, the grains of AA7075 become much coarser, which may be related to the heating of the solution treatment (Figure 2b). The grains easily grow under the high temperature of the solution treatment [25], and meanwhile, the strengthening precipitates (i.e., Guinier–Preston zones, η/η’-MgZn_2_ phases [26,27]) dissolve into the aluminum matrix, and only some Fe-Mn-Cr particles with high dissolution points remained (Figure 2c).

**Figure 3:**
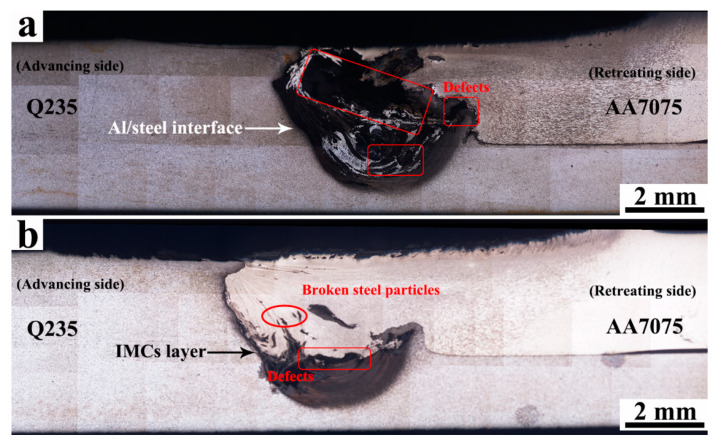
The overviews of dissimilar Al/steel butt-lap joints fabricated via (**a**) FSW and (**b**) UaFSW.

The overviews of a dissimilar Al/steel butt-lap joint fabricated by FSW and UaFSW joints are shown in Figure 3, which exhibit three features: (I) the initially straight Al/steel interface becomes a curve due to the stirring of the tool; (II) the broken steel particles distribute in the NZ of the aluminum matrix; and (III) an upward steel hook inserts into the AA7075 plate. Besides this, some welding defects (i.e., tunnel defects) are also detected in the NZ of the FSW joint (Figure 3a), which are caused by the relatively low welding heat input used in this study. The low welding heat input weakens the material flow during FSW, and thus the cavities left by the stir tool cannot be filled adequately, resulting in the formation of tunnel defects [28]. By contrast, the application of ultrasonic vibration leads to the enhancement of the material flow, and therefore, the volume of welding defects in the UaFSW joint decreases significantly (Figure 3b).

**Figure 4:**
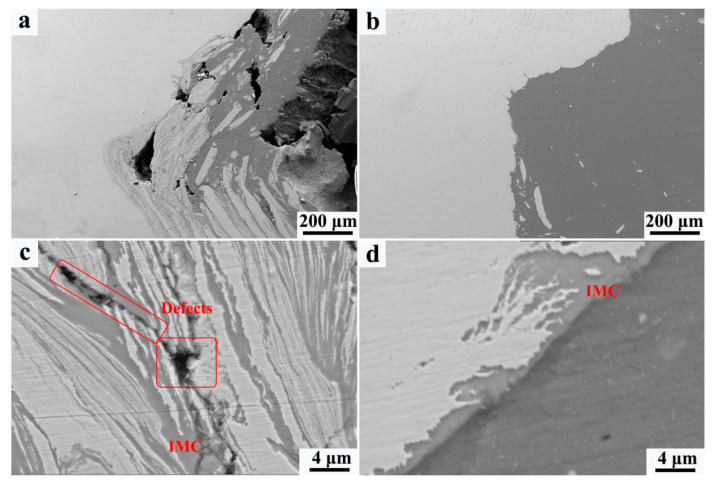
Low-magnification SEM of Al/steel interface in the (**a**) FSW and (**b**) UaFSW joint; the morphology of IMCs layer in the (**c**) FSW and (**d**) UaFSW joint.

**Figure 5:**
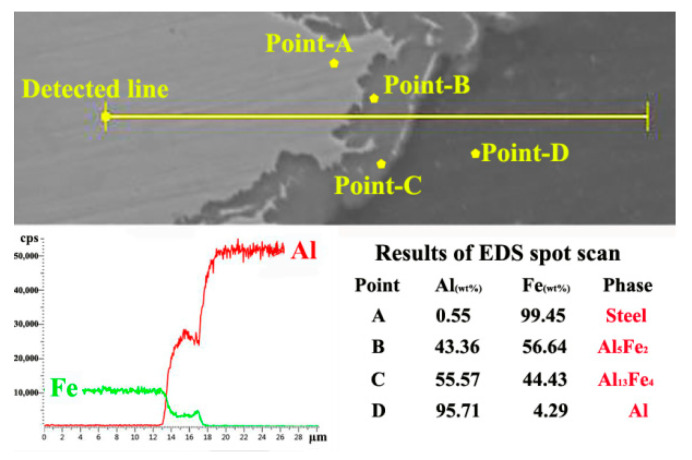
The results of EDS line scan and spot scan through the IMCs layer of UaFSW joint.

The microstructural evolution of the Al/steel interface is analyzed by SEM, as shown in Figure 4, where the color of steel is white while that of aluminum is dark. For the FSW joint, the steel adjacent to the Al/steel interface is broken by the stir tool and gets mixed with aluminum (Figure 4a); however, the mixture of Al/steel is not sufficient and gives rise to the formation of welding defects. Differently, the Al/steel interface of the UaFSW joint is smooth and the sizes of broken steel particles become finer (Figure 4b). This phenomenon can be explained by the acoustoplastic effect of ultrasonic vibration [29,30]: the energy of ultrasonic vibration could remarkably lead to the softening of metallic materials, and the influence of the reduction in the flow stress/yield stress of metallic materials by high-frequency vibration is called the acoustoplastic effect. The Q235 becomes softer because of the acoustoplastic effect, and hence the stir tool breaks the steel into pieces easier and transfers the broken steel particles further away from the Al/steel interface. The IMCs layer forms along the Al/steel interface for both the FSW and UaFSW joints, as shown in Figure 4c,d. For the FSW joint, the IMCs layer is striped and thick, moreover, small defects (i.e., cavities) can also be found between the Al and steel. By comparison, the IMCs layer of the UaFSW joint becomes continuous and the average thickness is less than 8 μm. The thinner IMCs layer is beneficial for the strengthening of the UaFSW joint. The Al/Fe element distribution through the IMCs layer of the UaFSW joint is analyzed by an EDS line scan (Figure 5): the changing trend of the Al/Fe elements is relatively steady, forming a 5 μm-thick Al/Fe IMCs layer. Based on the results of the EDS spot scan, the IMCs layer possibly consists of Al_5_Fe_2_ and Al_13_Fe_4_ phases. The above inference can also be supported by references [1,31], which reported that the Al_13_Fe_4_ phase was firstly generated and then transformed into the Al_5_Fe_2_ phase during FSW.

**Figure 6:**
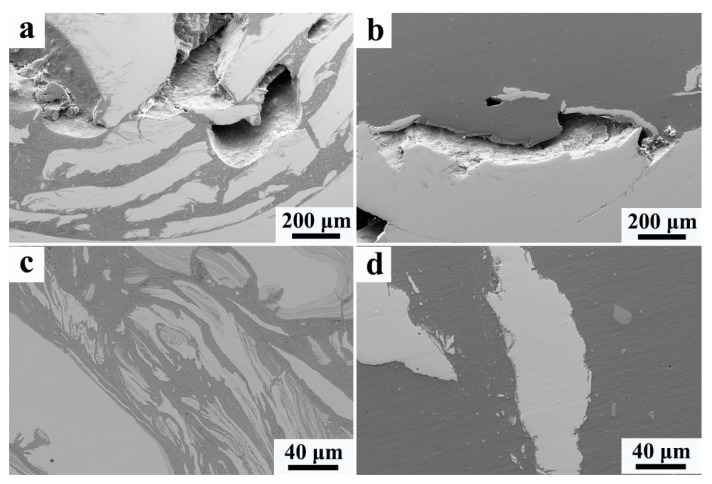
The welding defects of (**a**) FSW and (**b**) UaFSW joint and the broken steel particles in the (**c**) FSW and (**d**) UaFSW joint observed at the bottom of the NZ.

**Figure 7:**
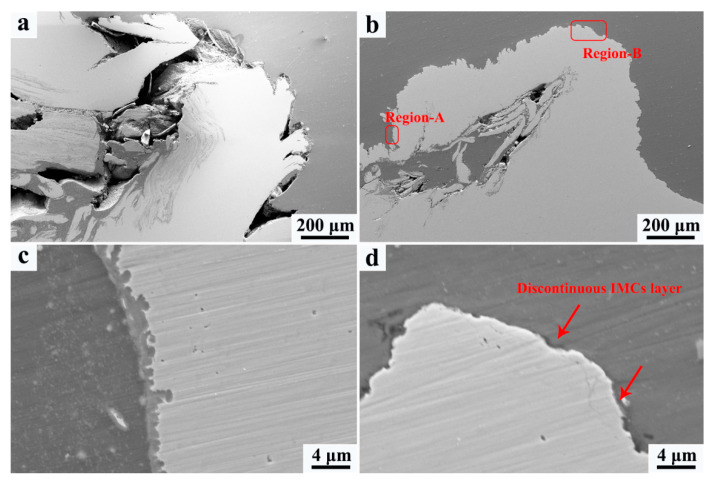
The welding defects around the steel hook of (**a**) FSW and (**b**) UaFSW joint, (**c**) thin IMCs layers in region-A, (**d**) discontinuous IMCs layer in region-B.

At the bottom of the NZ, the welding defects are detected in both the FSW and UaFSW joints (Figure 6a,b), indicating that the effect of the ultrasonic vibration becomes negligible in this region. This might be related to the low welding temperature at the bottom of the NZ. The heat input caused by the shoulder decreases due to the thermal transmission of the lower steel plate [32], and the acoustoplastic effect of the ultrasonic vibration cannot counteract the hardening increment caused by the cooling. Consequently, the material flow gets weakened and gives rise to the formation of a welding defect. Meanwhile, the tool cannot stir the steel adequately and the steel particles become large at the bottom of the NZ (Figure 6c,d). The reduced acoustoplastic effect can also be observed around the steel hook structure, where the cavities form in both the FSW and UaFSW joints (Figure 7a,b). In addition, a thin IMCs layer is detected at the Al/steel interface of the hook in the UaFSW joint (Figure 7c); however, the discontinuous IMCs layer indicates that the bonding between the Al and steel is weak (Figure 7d).

**Figure 8:**
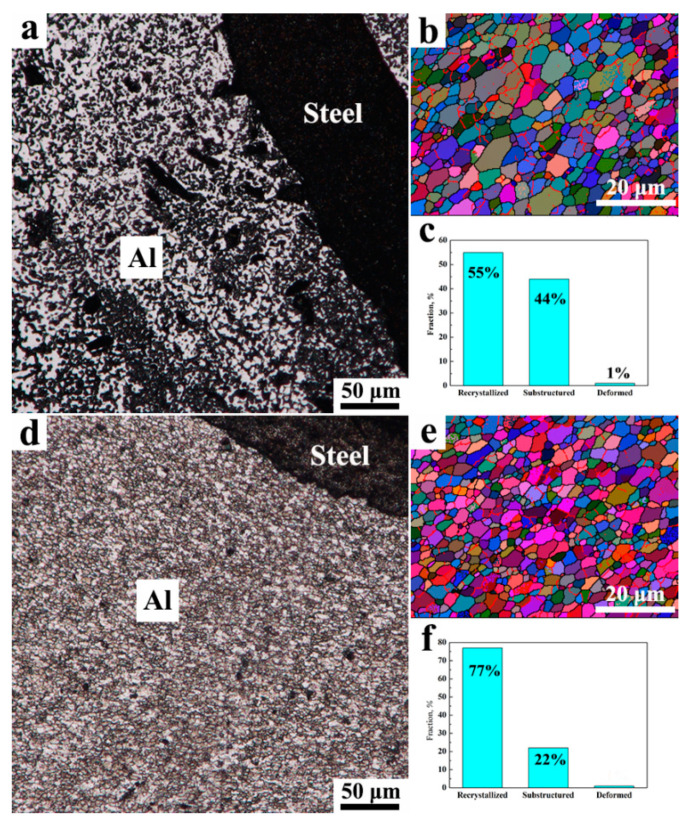
The grain morphology in the NZ of (**a**) FSW and (**d**) UaFSW joint observed by OM; the grain information in the NZ of (**b**) FSW and (**e**) UaFSW joint analyzed by EBSD; the recrystallization fraction in the NZ of (**c**) FSW and (**f**) UaFSW joint.

The NZ of the joint is mainly located in the aluminum side due to the offset of the stir tool and, meanwhile, a small amount of steel is stirred into the aluminum matrix, acting as the strengthening particles. The grain morphology of aluminum in the NZ is shown in Figure 8a,d; fine and equiaxed grains take the place of initial coarse grains because of the dynamic recrystallization (DRX) during FSW [33]. For the FSW joint, the average grain size of aluminum is 3.2 μm (Figure 8b) and the fraction of recrystallization is 55% (Figure 8c). In comparison, the grains get finer in the UaFSW joint, which is 2.5 μm on average (Figure 8e). Moreover, the recrystallization of the UaFSW joint becomes much more sufficient and a 22% increment in the fraction of recrystallization is found (Figure 8f). Azimzadegan et al. [34] stated that the final grain size of DRX could be calculated as Equation (1):

(1) $$D^{-1}=a+b\ln[\dot{\varepsilon }\exp(\frac{Q}{RT})]$$

where *D* is the final grain diameter of DRX, *Q* is the activation energy, $\dot{\varepsilon }$ is the strain rate, *T* is the welding temperature, and *a*, *b,* and *R* are constants. Thus, it can be inferred that fine grains can be obtained using a low welding temperature or high strain rate. Shi et al. [35] stated that the thermal effect of ultrasonic vibration on FSW was slight and the superimposed ultrasonic vibration caused little increases in the welding temperature. By contrast, the utilization of ultrasonic vibration leads to a decrease in the viscosity of materials, increasing both the material flow velocity and strain rate [36,37]. Therefore, in comparison to the FSW joint, the finer grains of the UaFSW joint are attributed to the higher strain rate caused by the ultrasonic vibration. Besides, the DRX grain size can also be calculated by Equation (2):

(2) $$D=C(\frac{G}{N_{A}}){]}^{1/3}$$

where *C* is the materials constant, *N_A_* is the nucleation rate, and *G* is the growth rate. It has been reported that both *N_A_* and *G* increased with the increase of the stored energy, and the stored energy had a more marked effect on the former than the latter [38]. Thus, finer grains can also be obtained by increasing the stored energy, and the application of ultrasonic vibration led to the elevation of stored energy, refining the grains in the NZ of the UaFSW joint. The reason for the different DRX evolutions between FSW and UaFSW is complex, which may be related to the migration of dislocations/grain boundaries. Hu et al. [39] reported that the energy of ultrasonic vibration accelerated both the dislocation climb and the grain boundaries mobility, promoting the DRX during UaFSW.

**Figure 9:**
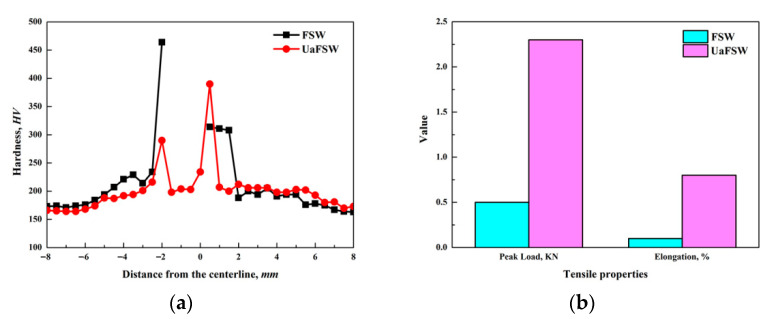
(**a**) The hardness distribution through the cross section of FSW and UaFSW joint, (**b**) the tensile properties of FSW and UaFSW joint.

Figure 9a shows the hardness distribution through the cross section of FSW and UaFSW joints, and it can be seen that the hardness gets increased and becomes fluctuant in the NZ. Based on the microstructural observations (as shown from Figure 3, Figure 4, Figure 5, Figure 6, Figure 7 and Figure 8), the NZ is comprised of a refined aluminum matrix and broken steel particles, which induces high levels of both grain boundary-strengthening and dispersion-strengthening [40], elevating the hardness of the NZ. However, the distribution of broken steel particles in the NZ is not uniform enough, resulting in the fluctuant of hardness. The overall mechanical performance of the joints is evaluated by the tensile test, and the UaFSW joint shows better tensile properties in comparison to the FSW joint (Figure 9b): the peak tensile load of the FSW joint is only 0.8 KN, while it increases to 2.3 KN for the UaFSW joint. Similarly, a more than 200% increment is obtained in the elongation of the UaFSW joint.

**Figure 10:**
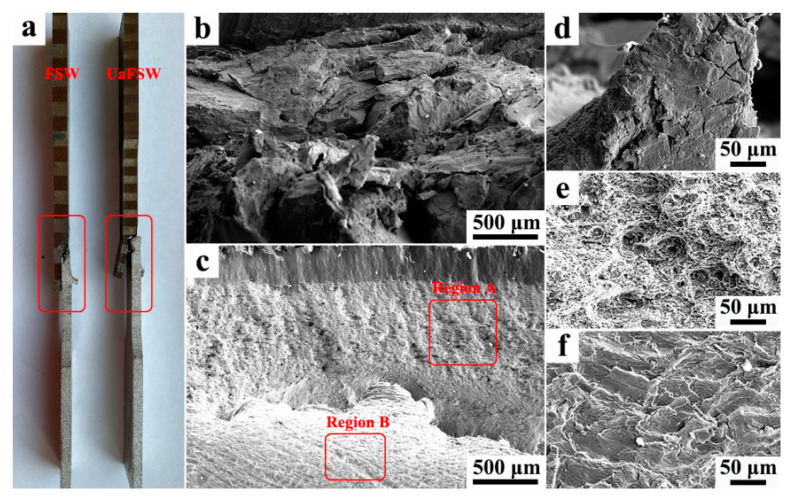
(**a**) The image of failure tensile specimens, the fracture surface of (**b**,**d**) FSW joint, and (**c**) UaFSW joint; the magnified SEM images of (**e**) region A and (**f**) region B in the UaFSW joint.

The images of failure specimens are shown in Figure 10a, it can be seen that all the specimens fail in the NZ while the failure paths are different for the FSW and UaFSW joints. For the FSW joint, the crack initiates from the welding defects and the tensile specimen fails rapidly without necking, indicating that the elongation of FSW joint is slight. In comparison, the failure occurs along the Al/steel interface for the UaFSW joint, and the enhanced plastic deformation leads to the bending of the steel plate. Figure 10b exhibits the fracture surface of the FSW joint; no dimples were detected and only the large aluminum and steel blocks without plastic deformation can be observed (Figure 10d). The existence of welding defects destroys the bonding between Al and steel, and it becomes easy to pull Al and steel from each other [13]. Differently, the fracture surface of the UaFSW joint shows a mixture of ductile–brittle features (Figure 10c): numerous ductile dimples distribute at the top of the NZ (Figure 10e) and, meanwhile, the fracture surface at the bottom of the NZ becomes flat (Figure 10f). The IMCs layer at the top of the NZ is smooth and thin (as shown in Figure 4b,d), which provides an effective bonding between Al and steel. Nevertheless, the formation of welding defects and discontinuous IMC layers deteriorate the bonding quality at the bottom of the NZ (Figure 6 and Figure 7), which shifts the fracture mode from ductile to brittle.

It is still difficult to obtain a sound Al/steel butt-lap joint via UaFSW at this time, and much work is needed to be done in the future. The authors propose one possible way to elevate the quality of the Al/steel butt-lap joint: the energy input of a single ultrasonic source may be not sufficient enough for the butt-lap structure, and thus, a doubling or tripling of ultrasonic sources can be employed in order to increase the energy input.

## 4. Conclusions

The microstructural/mechanical evolutions of dissimilar Al/steel butt-lap joints fabricated via UaFSW were investigated in the present study, and the following results can be summarized:

(1) Compared with FSW, the material flow during UaFSW was enhanced, decreasing the volume of welding defects in the NZ. Moreover, the IMCs layers at the top of the NZ became smooth and thin due to the acoustoplastic effect of ultrasonic vibration, and the IMCs layer was mainly comprised of Al_5_Fe_2_ and Al_13_Fe_4_ phases. However, the acoustoplastic effect was weakened at low temperatures. As a result, both welding defects and discontinuous IMCs layers formed at the bottom of the NZ;
(2) The application of ultrasonic vibration accelerated the DRX process, and the fraction of recrystallization increased from 55% to 77%. Besides, superimposing ultrasonic vibration led to better grain refinement in the NZ, which was caused by the increased strain rate and stored energy. In comparison to the FSW joint, the tensile properties of the UaFSW joint were elevated, and a 1.5 KN (or 200%) increment in the peak tensile load (or elongation) was obtained for the UaFSW joint. Meanwhile, the fracture mode of the UaFSW joint shifted from ductile at the top of the NZ to brittle at the bottom of the NZ.

## Figures and Tables

**Table 1:** Nominal chemical compositions (wt. %) of studied AA7075 aluminum and Q235 steel.

**AA7075**	**Zn**	**Mg**	**Cu**	**Mn**	**Fe**	**Cr**	**Al**
	5.72	2.36	1.65	0.22	0.31	0.24	Bal
**Q235**	**C**	**Si**	**Mn**	**P**	**S**	**(Nb-Al-V)**	**Fe**
	0.13	0.03	0.43	0.03	0.02	~0.01	Bal

## Data Availability

Not applicable.
